# 新生儿纵隔巨大畸胎瘤术后膈神经麻痹

**DOI:** 10.3779/j.issn.1009-3419.2015.08.11

**Published:** 2015-08-20

**Authors:** 远大 程, 燕 艾, 阳 高, 春芳 张

**Affiliations:** 410008 长沙，中南大学湘雅医院胸外科 Department of Thoracic Surgery, Xiangya Hospital, Central South University, Changsha 410008, China

**Keywords:** 新生儿, 纵隔, 畸胎瘤, 膈神经麻痹, Neonate, Mediastinum, Teratoma, Phrenic nerve palsy

## Abstract

新生儿畸胎瘤临床上不多见，多为病例报道，女性多于男性，大多数为良性，可发生于身体中线的任何部位，骶尾部为最好发部位，纵隔是性腺以外的第二大好发部位。临床上新生儿畸胎瘤多为良性，恶性少见，外科完整切除是其主要治疗方式。本文报道1例新生儿畸胎瘤，其术后发生致死性膈神经麻痹。

## Abstract (en)

Neonatal teratomas, not common in clinical, are often some case reports, female more than male, most are benign. It can occur anywhere of body midline; sacrococcygeal teratoma is the most common and the second most frequent site of extragonadal teratomas is mediastinum. Benign is more commom and malignant is very rarely seen. Completely surgical resection is the main and effective treatment. This review reports a case of neonatal teratoma, which is complicated with a fatal phrenic nerve palsy after surgery.

## 1 临床资料

新生儿，女，孕40^+1^周顺产出生，因出生后呼吸困难1 h收治我院新生儿科。入院体查：心率146次/分，呼吸70次/分，血压73/50 mmHg，体重3, 080 g，胸廓基本对称，气管右偏，左上肺呼吸音偏低，右侧胸骨旁心音明显。其母亲孕38周彩超检查提示宫内胎儿左侧胸腔内占位性病变。出生后计算机断层扫描（computed tomography, CT）提示左前纵隔软组织影，大小约4 cm×6 cm，平扫CT值约14 Hu，增强后不均匀强化，CT值约42 Hu，纵隔受压右移（图 1A，图 1B）。

**1:**
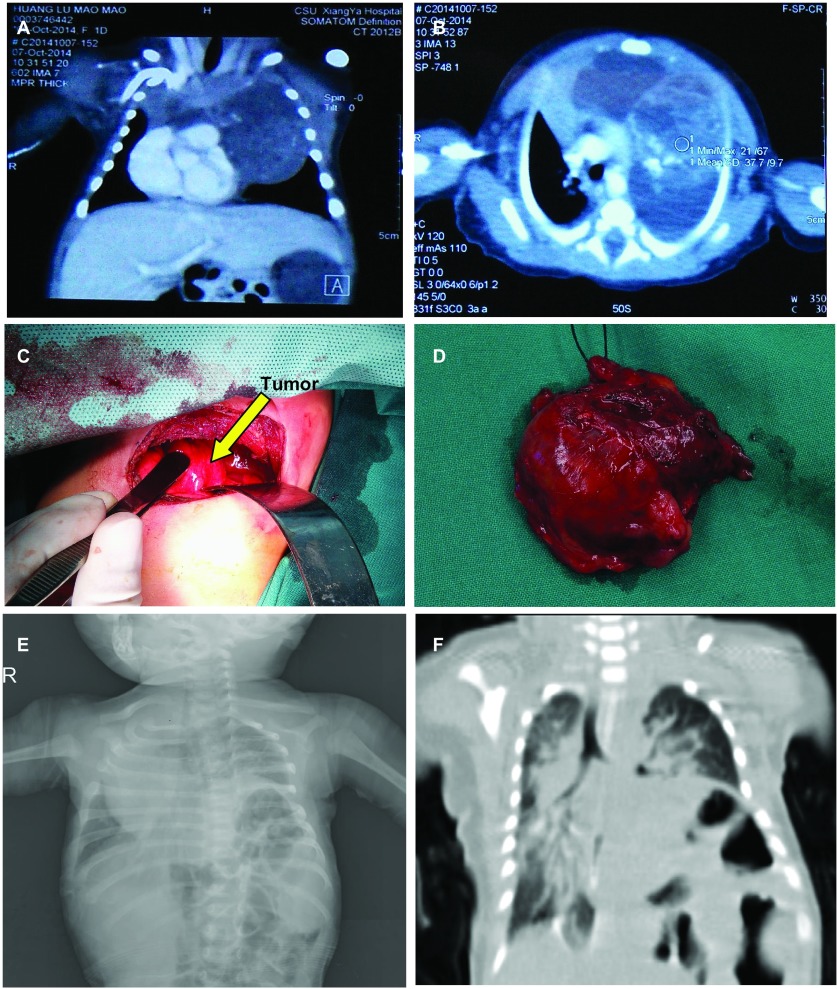
患者影像学图片。A、B为术前患儿CT，可见前纵隔巨大占位，纵隔明显右移，肿瘤内密度不均匀；C为术中图片，D为完整切除的肿瘤；E、F为术后复查的胸片和CT，可见纵膈仍然向右移位，左侧膈肌明显抬高。 Pictures of patient. A and B are preoperative CT scan, which show huge anterior mediastial tumor with different Hu on CT and mediastium shifts to the right. C is in operation and D is the tumor removed completely. E and F are the postoperative X-ray and CT, from which mediastium still shifts to right and left diaphragm rises up obviously. CT: computed tomography.

为解除压迫症状，于2014年10月13日患儿出生后第7天接受了开胸手术。手术沿左侧第4肋床行外侧切口进胸（图 1C），术中见：肿块大小约6 cm，侵犯壁层胸膜、胸腺，手术切除受累胸膜，保护胸腺、头臂静脉及上腔静脉，完整切除肿块及周围脂肪组织（图 1D）。

术后病检结果示：未成熟畸胎瘤2级。术后患儿呼吸困难症状改善，间断有呼吸困难情况，经积极抗感染、呼吸机辅助通气等对症支持治疗，于术后1个月出院，出院后2天，患儿出现呕吐、呼吸困难加重再次入院。体查：吸气时三凹症，左下肺呼吸音低，双肺闻及少许啰音。复查胸片（图 1E）和CT（图 1F）示双肺感染，左侧膈肌上抬明显，纵隔向右侧移位明显，考虑纵隔疝，临床考虑为术后左侧膈神经麻痹引起。经内科积极治疗，患儿病情稍好转，于2014年11月26日出院。出院后1个月随访，患儿因呼吸衰竭已死亡。

## 2 讨论

该病例具有一定的特殊性，临床上非常罕见：①畸胎瘤巨大，肿瘤大小约6 cm，由影像学检查可见肿瘤占据了左侧大部分胸腔，纵隔受压右移；②病检为未成熟2级，临床并不多见，新生儿纵隔畸胎瘤临床上良性成熟畸胎瘤多见；③患儿年龄较小，出生后7天即接受手术治疗；④患儿术后出现致死性的并发症，即膈神经麻痹致严重的呼吸循环衰竭。

新生儿畸胎瘤，女性多于男性，大多数为良性，其发病机制目前尚不完全清除，常发生于从颅脑至骶尾部的身体中线的任何部位，其中骶尾部最为常见，纵隔是性腺以外的新生儿畸胎瘤第二大好发部位^[1]^。新生儿纵隔畸胎瘤约占新生儿畸胎瘤的7%左右，约85%为良性^[2]^，目前临床上多为病例报道^[3-5]^。新生儿纵隔畸胎瘤可在产前检查时被发现，妇科B超常提示胎儿胸腔内占位。少数前纵隔畸胎瘤因生长迅速，对心肺产生严重的压迫，常合并非免疫性水肿胎儿（non-immune hydrops fetalis, NIHF）。至目前仅有5例有关纵隔畸胎瘤合并NIFH的报道^[3]^。产后新生儿的CT或核磁共振成像（magnetic resonance imaging, MRI）对诊断提供较大的临床参考价值，但其确诊需靠病理检查。纵隔畸胎瘤的早期发现和诊断，对预后有重要意义。

对于新生儿纵隔畸胎瘤，尤其是肿瘤体积较大的，临床上常常需要外科手术切除。术式的选择应根据肿瘤的位置、大小选择正中开胸或侧开胸，对于一些小的病变可选择胸腔镜手术。但对于巨大的纵隔畸胎瘤应注意避免术中损伤一些重要的脏器，尤其是胸腺、头臂静脉和膈神经。该患儿因肿瘤巨大，根据术后复查情况，考虑术中左侧膈神经可能受到损伤进而出现膈神经麻痹症状。一侧膈神经的麻痹会导致膈肌的上抬，纵隔向健侧移位，严重者为纵隔疝，从而影响患者的呼吸、循环和消化系统，如膈肌上抬压迫引起肺不张，继发肺部感染，纵隔移位导致循环不稳定，膈肌上抬后腹腔的肠管移位甚至扭转可出现恶心呕吐等消化系统症状。

新生儿膈神经麻痹或损伤常见于出生时的产伤、纵隔或心脏的手术等。新生儿膈神经麻痹不同于幼儿或儿童，小儿膈神经切断后呼吸系统症状不明显，Gammal等^[7]^认为3岁以上患儿行膈神经移位术是安全的，因此膈神经离断、移位常用来治疗小儿臂丛神经损伤。但新生儿膈神经的麻痹或损伤常会导致严重的呼吸窘迫，呼吸系统后遗症，影响生长发育^[8, 9]^。早在20世纪90年代，Serraf等^[10]^认为新生儿及婴幼儿对膈神经损伤的耐受程度差，常需要机械通气且呼吸系统并发症多。杨等^[11]^的动物实验证实，年龄越小膈神经损伤对呼吸系统的影响越明显。小儿尤其是婴幼儿正处于出生后肺发育的关键阶段，膈神经切断后的膈肌麻痹将导致肺组织周期性牵张应力明显减弱甚至消失，进而影响肺发育引起肺组织功能及形态方面异常。目前对新生儿或幼儿膈神经麻痹或切断后的影响，研究较少，尚无统一的观点，总之，年龄越小，膈神经麻痹对机体的影响越大。由该病例可见，新生儿膈神经麻痹对呼吸、循环的影响极大，具有致死性。因此，在临床工作中，尤其是在婴幼儿的胸部手术中，我们应当注意保护膈神经避免受到损伤。

新生儿纵隔畸胎瘤无论良性和恶性均有复发的几率，因此定期随访非常重要。目前临床上常检查甲胎蛋白AFP作为畸胎瘤肿瘤标志物来评估肿瘤残留或复发^[1, 12]^。正常情况下，AFP在胎儿体内表达较高，但随着年龄增长，逐渐下降至正常水平。患有畸胎瘤的患儿，如手术切除彻底，其术后AFP水平下降明显。术后血清中持续高水平的AFP常提示肿瘤残留，可能需要再次手术或术后辅助化疗。
